# 4-Eth­oxy-*N*-(3-phenyl­prop-2-enyl­idene)aniline

**DOI:** 10.1107/S1600536808014050

**Published:** 2008-05-17

**Authors:** Yu-Ying Sun, Qiang Wang, Da-Qi Wang

**Affiliations:** a Analytical and Testing Center of Beihua Univerisity, Jilin 132031, People’s Republic of China; b College of Chemistry and Chemical Engineering, Liaocheng University, Shandong 252059, People’s Republic of China

## Abstract

The title compound, C_17_H_17_NO, was prepared by the condensation of cinnamaldehyde with *p*-phenetidine in ethanol. The prop-2-enyl­idene group exhibits an *E* configuration at the N=C and C=C double bonds, with C—N—C—C and C—C—C—C torsion angles of −179.9 (3) and −175.9 (3)°, respectively. The prop-2-enyl­idene group is not strictly planar [maximum deviation = 0.054 (4) Å] and forms dihedral angles of 28.0 (3) and 34.9 (3)° with the attached aromatic rings.

## Related literature

For general background, see: Lindoy *et al.* (1976 ▶).
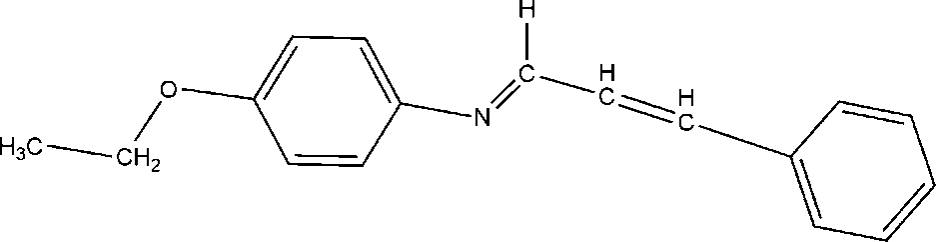


## Experimental

### 

#### Crystal data

C_17_H_17_NO
*M* *_r_* = 251.32
Monoclinic, 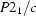

*a* = 31.12 (2) Å
*b* = 7.198 (6) Å
*c* = 6.315 (5) Å
β = 95.822 (10)°
*V* = 1407.3 (19) Å^3^
*Z* = 4
Mo *K*α radiation
μ = 0.07 mm^−1^
*T* = 298 (2) K
0.52 × 0.47 × 0.30 mm

#### Data collection

Siemens SMART CCD area-detector diffractometer
Absorption correction: multi-scan (*SADABS*; Sheldrick, 1996 ▶) *T*_min_ = 0.963, *T*_max_ = 0.978
6773 measured reflections
2449 independent reflections
1165 reflections with *I* > 2σ(*I*)
*R*_int_ = 0.072

#### Refinement

*R*[*F* ^2^ > 2σ(*F* ^2^)] = 0.076
*wR*(*F* ^2^) = 0.221
*S* = 1.02
2449 reflections
172 parameters
H-atom parameters constrained
Δρ_max_ = 0.17 e Å^−3^
Δρ_min_ = −0.35 e Å^−3^

### 

Data collection: *SMART* (Siemens, 1996 ▶); cell refinement: *SAINT* (Siemens, 1996 ▶); data reduction: *SAINT*; program(s) used to solve structure: *SHELXS97* (Sheldrick, 2008 ▶); program(s) used to refine structure: *SHELXL97* (Sheldrick, 2008 ▶); molecular graphics: *SHELXTL* (Sheldrick, 2008 ▶); software used to prepare material for publication: *SHELXTL*.

## Supplementary Material

Crystal structure: contains datablocks I, global. DOI: 10.1107/S1600536808014050/rz2203sup1.cif

Structure factors: contains datablocks I. DOI: 10.1107/S1600536808014050/rz2203Isup2.hkl

Additional supplementary materials:  crystallographic information; 3D view; checkCIF report

## Figures and Tables

**Fig. 1.**
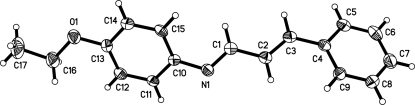
The molecular structure of the title compound, showing 30% probability displacement ellipsoids and the atom-numbering scheme.

**Fig. 2.**
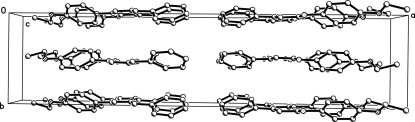
Perspective view of the crystal packing of the title compound along the c axis. Hydrogen atoms are omitted for clarity.

## supplementary crystallographic information

### Comment

Schiff bases are known to be important due to their applications in the synthesis of dyes, liquid crystals and as powerful corrosion inhibitors. Furthermore, they are involved in the mechanisms of many biochemical processes (Lindoy *et al.*, 1976). We report here the synthesis and crystal structure of the title compound, a new Schiff base compound.

The molecular structure of the title compound is shown in Fig. 1. The prop-2-enylidene group exhibits an E configuration at the N1═C1 (1.276 (4) Å) and C2═C3 (1.321 (5) Å) double bonds, with C10-N1-C1-C2 and C1-C2-C3-C4 torsion angles of -179.9 (3)° and -175.9 (3)° respectively. This group is not strictly planar (maximum deviation 0.054 (4) Å for atom C2) and forms dihedral angles of 28.0 (3) and 34.9 (3)° with the attached aromatic rings. The crystal structure (Fig. 2) is stabilized only by van der Waals interactions.

### Experimental

Cinnamaldehyde (5 mmol, 660.8 mg) in absolute ethanol (10 ml) was added dropwise to an absolute ethanol solution (10 ml) of *p*-phenetidine (5 mmol, 690.7 mg). The mixture was heated under reflux with stirring for 4 h and then filtered. The resulting clear solution was kept at room temperature for one week, after which large pale-yellow block-shaped crystals of the title compound suitable for X-ray diffraction analysis were obtained.

### Refinement

All H-atoms were positioned geometrically and refined using a riding model, with C—H = 0.93-0.97 Å, and *U*_iso_(H) =1.2*U*_eq_(C) or 1.5*U*_eq_(C) for methyl H atoms.

### Crystal data

**Table:**

C_17_H_17_NO	*F*_000_ = 536
*M**_r_* = 251.32	*D*_x_ = 1.186 Mg m^−^^3^
Monoclinic, *P*2_1_/*c*	Mo *K*α radiation λ = 0.71073 Å
Hall symbol: -P 2ybc	Cell parameters from 1073 reflections
*a* = 31.12 (2) Å	θ = 2.6–23.2º
*b* = 7.198 (6) Å	µ = 0.07 mm^−^^1^
*c* = 6.315 (5) Å	*T* = 298 (2) K
β = 95.822 (10)º	Block, pale-yellow
*V* = 1407.3 (19) Å^3^	0.52 × 0.47 × 0.30 mm
*Z* = 4	

### Data collection

**Table:**

Siemens SMART CCD area-detector diffractometer	2449 independent reflections
Radiation source: fine-focus sealed tube	1165 reflections with *I* > 2σ(*I*)
Monochromator: graphite	*R*_int_ = 0.072
*T* = 298(2) K	θ_max_ = 25.0º
φ and ω scans	θ_min_ = 2.0º
Absorption correction: multi-scan(SADABS; Sheldrick, 1996)	*h* = −36→37
*T*_min_ = 0.963, *T*_max_ = 0.978	*k* = −8→7
6773 measured reflections	*l* = −7→5

### Refinement

**Table:**

Refinement on *F*^2^	Secondary atom site location: difference Fourier map
Least-squares matrix: full	Hydrogen site location: inferred from neighbouring sites
*R*[*F*^2^ > 2σ(*F*^2^)] = 0.076	H-atom parameters constrained
*wR*(*F*^2^) = 0.221	*w* = 1/[σ^2^(*F*_o_^2^) + (0.0854*P*)^2^ + 0.6793*P*] where *P* = (*F*_o_^2^ + 2*F*_c_^2^)/3
*S* = 1.02	(Δ/σ)_max_ < 0.001
2449 reflections	Δρ_max_ = 0.17 e Å^−^^3^
172 parameters	Δρ_min_ = −0.35 e Å^−^^3^
Primary atom site location: structure-invariant direct methods	Extinction correction: none

### Special details

**Table:**

Geometry. All e.s.d.'s (except the e.s.d. in the dihedral angle between two l.s. planes) are estimated using the full covariance matrix. The cell e.s.d.'s are taken into account individually in the estimation of e.s.d.'s in distances, angles and torsion angles; correlations between e.s.d.'s in cell parameters are only used when they are defined by crystal symmetry. An approximate (isotropic) treatment of cell e.s.d.'s is used for estimating e.s.d.'s involving l.s. planes.
Refinement. Refinement of *F*^2^ against ALL reflections. The weighted *R*-factor *wR* and goodness of fit *S* are based on *F*^2^, conventional *R*-factors *R* are based on *F*, with *F* set to zero for negative *F*^2^. The threshold expression of *F*^2^ > σ(*F*^2^) is used only for calculating *R*-factors(gt) *etc*. and is not relevant to the choice of reflections for refinement. *R*-factors based on *F*^2^ are statistically about twice as large as those based on *F*, and *R*- factors based on ALL data will be even larger.

### Fractional atomic coordinates and isotropic or equivalent isotropic displacement parameters (Å^2^)

**Table:**

	*x*	*y*	*z*	*U*_iso_*/*U*_eq_	
N1	0.26172 (9)	0.5173 (4)	0.8270 (5)	0.0442 (8)	
O1	0.09317 (8)	0.5040 (4)	1.0522 (4)	0.0547 (8)	
C1	0.27064 (12)	0.5295 (5)	0.6347 (6)	0.0434 (10)	
H1	0.2481	0.5343	0.5262	0.052*	
C2	0.31443 (12)	0.5359 (5)	0.5801 (6)	0.0452 (10)	
H2	0.3364	0.5506	0.6903	0.054*	
C3	0.32565 (12)	0.5225 (5)	0.3845 (6)	0.0450 (10)	
H3	0.3033	0.5162	0.2750	0.054*	
C4	0.36937 (11)	0.5166 (5)	0.3235 (6)	0.0410 (10)	
C5	0.37720 (13)	0.4321 (6)	0.1308 (6)	0.0503 (11)	
H5	0.3542	0.3854	0.0408	0.060*	
C6	0.41851 (14)	0.4178 (6)	0.0742 (6)	0.0588 (12)	
H6	0.4232	0.3602	−0.0532	0.071*	
C7	0.45320 (14)	0.4877 (6)	0.2034 (7)	0.0608 (12)	
H7	0.4811	0.4772	0.1643	0.073*	
C8	0.44578 (12)	0.5730 (6)	0.3907 (6)	0.0537 (11)	
H8	0.4688	0.6214	0.4788	0.064*	
C9	0.40471 (11)	0.5873 (5)	0.4489 (6)	0.0454 (10)	
H9	0.4004	0.6462	0.5762	0.054*	
C10	0.21832 (10)	0.5112 (5)	0.8727 (5)	0.0353 (9)	
C11	0.20976 (11)	0.4159 (5)	1.0554 (5)	0.0387 (9)	
H11	0.2323	0.3578	1.1382	0.046*	
C12	0.16848 (11)	0.4062 (5)	1.1158 (6)	0.0431 (10)	
H12	0.1633	0.3379	1.2357	0.052*	
C13	0.13480 (11)	0.4965 (5)	1.0008 (6)	0.0390 (9)	
C14	0.14323 (11)	0.5926 (5)	0.8180 (6)	0.0414 (9)	
H14	0.1208	0.6528	0.7370	0.050*	
C15	0.18419 (11)	0.5995 (5)	0.7561 (5)	0.0398 (9)	
H15	0.1891	0.6647	0.6337	0.048*	
C16	0.08474 (13)	0.4248 (7)	1.2501 (7)	0.0698 (14)	
H16A	0.1052	0.4718	1.3637	0.084*	
H16B	0.0876	0.2907	1.2451	0.084*	
C17	0.03980 (16)	0.4766 (9)	1.2899 (9)	0.119 (2)	
H17A	0.0333	0.4245	1.4229	0.179*	
H17B	0.0198	0.4293	1.1769	0.179*	
H17C	0.0374	0.6095	1.2955	0.179*	

### Atomic displacement parameters (Å^2^)

**Table:**

	*U*^11^	*U*^22^	*U*^33^	*U*^12^	*U*^13^	*U*^23^
N1	0.048 (2)	0.048 (2)	0.0374 (19)	0.0020 (15)	0.0063 (14)	−0.0004 (16)
O1	0.0566 (18)	0.057 (2)	0.0525 (18)	0.0042 (13)	0.0146 (13)	0.0112 (15)
C1	0.047 (2)	0.039 (3)	0.044 (2)	0.0006 (17)	0.0020 (18)	−0.0006 (19)
C2	0.050 (2)	0.045 (3)	0.041 (2)	−0.0010 (18)	0.0020 (18)	0.0020 (19)
C3	0.049 (2)	0.042 (3)	0.042 (2)	−0.0004 (18)	−0.0036 (18)	0.0033 (19)
C4	0.051 (2)	0.035 (2)	0.037 (2)	0.0016 (17)	0.0039 (18)	0.0060 (18)
C5	0.064 (3)	0.049 (3)	0.037 (2)	−0.003 (2)	0.003 (2)	−0.001 (2)
C6	0.084 (3)	0.052 (3)	0.043 (3)	0.008 (2)	0.020 (2)	0.001 (2)
C7	0.059 (3)	0.068 (3)	0.057 (3)	0.009 (2)	0.016 (2)	0.011 (3)
C8	0.046 (2)	0.063 (3)	0.052 (3)	0.0026 (19)	0.0015 (19)	0.001 (2)
C9	0.046 (2)	0.046 (3)	0.044 (2)	0.0013 (18)	0.0048 (18)	−0.0039 (19)
C10	0.040 (2)	0.028 (2)	0.037 (2)	0.0033 (15)	0.0005 (16)	−0.0010 (17)
C11	0.046 (2)	0.038 (2)	0.031 (2)	0.0041 (16)	0.0019 (16)	0.0030 (17)
C12	0.057 (3)	0.036 (2)	0.036 (2)	0.0005 (18)	0.0066 (18)	0.0039 (18)
C13	0.041 (2)	0.035 (2)	0.042 (2)	−0.0028 (17)	0.0089 (18)	−0.0037 (18)
C14	0.050 (2)	0.035 (2)	0.039 (2)	0.0034 (17)	0.0006 (17)	−0.0016 (18)
C15	0.059 (2)	0.030 (2)	0.031 (2)	0.0018 (17)	0.0074 (17)	0.0044 (17)
C16	0.064 (3)	0.083 (4)	0.066 (3)	−0.001 (2)	0.022 (2)	0.016 (3)
C17	0.083 (4)	0.168 (7)	0.116 (5)	0.020 (4)	0.057 (3)	0.049 (5)

### Geometric parameters (Å, °)

**Table:**

N1—C1	1.276 (4)	C8—H8	0.9300
N1—C10	1.410 (4)	C9—H9	0.9300
O1—C13	1.368 (4)	C10—C15	1.384 (5)
O1—C16	1.422 (4)	C10—C11	1.391 (4)
C1—C2	1.440 (5)	C11—C12	1.378 (4)
C1—H1	0.9300	C11—H11	0.9300
C2—C3	1.321 (5)	C12—C13	1.376 (5)
C2—H2	0.9300	C12—H12	0.9300
C3—C4	1.452 (5)	C13—C14	1.393 (5)
C3—H3	0.9300	C14—C15	1.372 (4)
C4—C9	1.386 (5)	C14—H14	0.9300
C4—C5	1.404 (5)	C15—H15	0.9300
C5—C6	1.373 (5)	C16—C17	1.493 (6)
C5—H5	0.9300	C16—H16A	0.9700
C6—C7	1.380 (6)	C16—H16B	0.9700
C6—H6	0.9300	C17—H17A	0.9600
C7—C8	1.373 (5)	C17—H17B	0.9600
C7—H7	0.9300	C17—H17C	0.9600
C8—C9	1.369 (5)		
			
C1—N1—C10	120.1 (3)	C15—C10—N1	125.2 (3)
C13—O1—C16	117.2 (3)	C11—C10—N1	117.0 (3)
N1—C1—C2	122.2 (3)	C12—C11—C10	121.1 (3)
N1—C1—H1	118.9	C12—C11—H11	119.4
C2—C1—H1	118.9	C10—C11—H11	119.4
C3—C2—C1	124.6 (4)	C13—C12—C11	120.7 (3)
C3—C2—H2	117.7	C13—C12—H12	119.6
C1—C2—H2	117.7	C11—C12—H12	119.6
C2—C3—C4	126.4 (4)	O1—C13—C12	125.6 (3)
C2—C3—H3	116.8	O1—C13—C14	116.1 (3)
C4—C3—H3	116.8	C12—C13—C14	118.3 (3)
C9—C4—C5	117.2 (3)	C15—C14—C13	120.9 (3)
C9—C4—C3	123.3 (3)	C15—C14—H14	119.6
C5—C4—C3	119.5 (3)	C13—C14—H14	119.6
C6—C5—C4	120.5 (4)	C14—C15—C10	121.1 (3)
C6—C5—H5	119.8	C14—C15—H15	119.4
C4—C5—H5	119.8	C10—C15—H15	119.4
C5—C6—C7	121.1 (4)	O1—C16—C17	107.9 (4)
C5—C6—H6	119.5	O1—C16—H16A	110.1
C7—C6—H6	119.5	C17—C16—H16A	110.1
C8—C7—C6	118.8 (4)	O1—C16—H16B	110.1
C8—C7—H7	120.6	C17—C16—H16B	110.1
C6—C7—H7	120.6	H16A—C16—H16B	108.4
C9—C8—C7	120.6 (4)	C16—C17—H17A	109.5
C9—C8—H8	119.7	C16—C17—H17B	109.5
C7—C8—H8	119.7	H17A—C17—H17B	109.5
C8—C9—C4	121.8 (4)	C16—C17—H17C	109.5
C8—C9—H9	119.1	H17A—C17—H17C	109.5
C4—C9—H9	119.1	H17B—C17—H17C	109.5
C15—C10—C11	117.8 (3)		
			
C10—N1—C1—C2	−179.9 (3)	C1—N1—C10—C11	151.0 (3)
N1—C1—C2—C3	170.1 (4)	C15—C10—C11—C12	1.4 (5)
C1—C2—C3—C4	−175.9 (3)	N1—C10—C11—C12	178.5 (3)
C2—C3—C4—C9	−23.3 (6)	C10—C11—C12—C13	−2.4 (5)
C2—C3—C4—C5	155.2 (4)	C16—O1—C13—C12	5.5 (5)
C9—C4—C5—C6	1.4 (5)	C16—O1—C13—C14	−173.2 (3)
C3—C4—C5—C6	−177.2 (4)	C11—C12—C13—O1	−176.5 (3)
C4—C5—C6—C7	−0.7 (6)	C11—C12—C13—C14	2.1 (5)
C5—C6—C7—C8	−0.2 (6)	O1—C13—C14—C15	177.7 (3)
C6—C7—C8—C9	0.4 (6)	C12—C13—C14—C15	−1.0 (5)
C7—C8—C9—C4	0.3 (6)	C13—C14—C15—C10	0.1 (5)
C5—C4—C9—C8	−1.2 (5)	C11—C10—C15—C14	−0.3 (5)
C3—C4—C9—C8	177.3 (4)	N1—C10—C15—C14	−177.1 (3)
C1—N1—C10—C15	−32.2 (5)	C13—O1—C16—C17	170.5 (4)
